# Characterization of the EBV-Induced Persistent DNA Damage Response

**DOI:** 10.3390/v9120366

**Published:** 2017-12-01

**Authors:** Amy Y. Hafez, Micah A. Luftig

**Affiliations:** Department of Molecular Genetics and Microbiology, Center for Virology, Duke University School of Medicine, Durham, NC 27710, USA; amy.hafez@duke.edu

**Keywords:** EBV, DNA damage, persistent DDR, telomere, senescence, immortalization

## Abstract

Epstein-Barr virus (EBV) is an oncogenic herpesvirus that is ubiquitous in the human population. Early after EBV infection in vitro, primary human B cells undergo a transient period of hyper-proliferation, which results in replicative stress and DNA damage, activation of the DNA damage response (DDR) pathway and, ultimately, senescence. In this study, we investigated DDR-mediated senescence in early arrested EBV-infected B cells and characterized the establishment of persistent DNA damage foci. We found that arrested EBV-infected B cells exhibited an increase in promyelocytic leukemia nuclear bodies (PML NBs), which predominantly localized to markers of DNA damage, as well as telomeric DNA. Furthermore, arrested EBV-infected B cells exhibited an increase in the presence of telomere dysfunction-induced foci. Importantly, we found that increasing human telomerase reverse transcriptase (hTERT) expression with danazol, a drug used to treat telomere diseases, permitted early EBV-infected B cells to overcome cellular senescence and enhanced transformation. Finally, we report that EBV-infected B cells undergoing hyper-proliferation are more sensitive than lymphoblastoid cell lines (LCLs) to inhibition of Bloom syndrome-associated helicase, which facilitates telomere replication. Together, our results describe the composition of persistent DNA damage foci in the early stages of EBV infection and define key regulators of this barrier to long-term outgrowth.

## 1. Introduction

Epstein-Barr virus (EBV), an oncogenic γ-herpesvirus was the first human tumor virus to be discovered [1]. Typically, EBV establishes an asymptomatic latent infection; however, EBV infection is associated with development of several lymphoid and epithelial cell malignancies, including Burkitt’s lymphoma, Hodgkin’s lymphoma, post-transplant lymphoproliferative disease, and nasopharyngeal carcinoma [1]. While the majority of the human population is infected with EBV, malignancies primarily develop in immunocompromised patients as the strong cytotoxic T cell response of the immune system acts to control the infection [2]. Additional evidence has been reported to support the idea that other intrinsic responses beyond the immune system serve to further control EBV-mediated transformation.

In vitro infection of primary human B cells with EBV leads to transformation of B cells into indefinitely-proliferating lymphoblastoid cell lines (LCLs). However, EBV-mediated transformation efficiency is so low that only about 1% of infected B cells become immortalized [3,4]. It has been shown that EBV induces a transient period of hyper-proliferation early after infection by upregulating the viral latency proteins, EBNA2 and EBNA-LP, to induce the expression of pro-growth genes, thus allowing for entry into the cell cycle [5,6,7,8]. Rapid cellular proliferation often leads to DNA damage that is recognized by the DNA damage response (DDR). This hyper-proliferative period is associated with increased levels of replicative stress and DNA damage, which triggers activation of the DDR, an innate tumor suppressor pathway [5,9,10,11,12,13,14]. Typically, activation of the DDR leads to DNA repair coupled with a transient cell cycle arrest, or if the damage is too great or irreparable, apoptosis or cellular senescence. We have previously found that EBV-infected B cells suppress apoptosis and trigger a G1/S phase cellular arrest [15,16]. Specifically, cellular arrest was observed to occur in approximately 60–80% of infected B cells [5].

Cellular senescence was first described by Leonard Hayflick and Paul Moorhead in 1961 to be defined as a stable cell growth arrest [17]. Senescence can be induced by several factors, such as DNA damage, critically-shortened telomeres, and oncogene activation [18,19,20]. Oncogene-induced senescence (OIS) is a growth arrest initiated upon oncogene overexpression or inactivation of tumor suppressors, which promotes cell cycle exit [18,19,20]. OIS is maintained by certain hallmarks, including DNA replication stress, persistent DDR activation, and upregulation of the p53/p21 and p16/pRb pathways, which ultimately facilitate stable growth arrest [10,11,21,22]. While OIS is known to trigger irreversible growth arrest, recent evidence suggests that cells arrested in OIS can escape senescence by derepression of human telomerase reverse transcriptase (*hTERT*) expression [23]. Our prior work suggests that EBV infection induces OIS in early proliferating B cells, suppressing transformation of primary B cells into LCLs [24]. Early after EBV infection, B cells undergo replicative stress and DNA damage as observed by nuclear halo assays and activate ATM/Chk2 and ATR/Chk1 DDR pathways [5,9,25]. Infected cells that arrest after undergoing rapid proliferation exhibit an increase in markers of OIS, including H3K9me3 senescence-associated heterochromatic foci, upregulation of p16 and p21, and enhancement of p53 target gene expression [15,24]. Furthermore, early infected B cells have been shown to undergo metabolic stress in the form of depressed oxidative phosphorylation and limited purine nucleotide pools, which may contribute to increased replication stress and establishment of persistent DNA damage foci [9,24].

Depending on the severity or location of DNA damage, lesions may either induce transient growth arrest to allow time for damage to be repaired, or lesions may establish persistent DNA damage foci, constitutive DDR signaling and chronic p53 activation, which leads to permanent senescence [26,27,28,29,30,31]. Irreparable persistent DNA damage foci have been shown to associate with complex breaks or uncapped telomeres [32,33]. Several studies have documented the hallmarks of persistent DNA damage foci, which include accumulation of DDR mediators, association of promyelocytic leukemia nuclear bodies (PML NBs) to DDR, localization of DDR to telomeric DNA or telomere dysfunction-induced foci (TIFs), localization of PML NBs to telomeric DNA or alternative lengthening of telomeres-associated PML NBs (APBs), and lack of DNA repair proteins [32,33,34,35]. The Masucci group has shown the presence of APBs in pre-immortalized EBV-infected B cells, along with evidence of telomere dysfunction [34]. While there is accumulating evidence for EBV-induced OIS, it is unclear the role persistent DDR plays in establishing OIS in early-infected B cells, as well as the composition of persistent DDR foci in this population of B cells. Therefore, in this study, we characterize the formation of persistent DDR foci in senescent EBV-infected B cells and the regulators of this response impinging on B-cell transformation.

## 2. Materials and Methods 

### 2.1. Viruses and Cells 

EBV B95-8 virus was produced from the B95-8 Z-HT cell line, as previously described [36]. Buffy coats were obtained from normal donors through the Gulf Coast Regional Blood Center and peripheral blood mononuclear cells (PBMCs) were isolated using a Ficoll Histopaque-1077 gradient (Sigma, St. Louis, MO, USA #H8889). Primary human B cells were cultured in Roswell Park Memorial Institute medium (RPMI)1640 supplemented with 15% fetal bovine serum (FBS), 2 mM l-glutamine, penicillin, and streptomycin (1X, Sigma, #G6784) (R15) as well as 0.5 µg/mL Cyclosporin A (CsA) (Sigma, #30024). Bulk infections were performed by incubating cells with B95-8 Z-HT supernatant at 500 μL per 10^6^ B cells calculated from within the PBMC population for 1 h at 37 °C in a CO_2_ incubator. Incubation was followed by washing in PBS and resuspending in R15 media supplemented with CsA. Bulk infections were conducted on 5 × 10^8^ PBMCs. LCLs were generated from normal donors by continuous outgrowth of EBV-infected primary B cells for greater than 35 days. LCLs were cultured in RPMI media supplemented with 10% FBS (R10). Bloom syndrome (BLM) deficient LCLs (GM16377, GM16375, and GM09960) were obtained from Coriell Institute (Coriell Institute, Camden, NJ, USA).

### 2.2. Chemicals

Bleomycin sulfate (Selleckchem, Houston, TX, USA, #S1214) was resuspended at 100 μg/mL in dimethyl sulfoxide (DMSO) and used to induce persistent DNA damage in LCLs at 20 μg/mL for two hours. Danazol (Sigma, #D8399) and ML216 BLM helicase inhibitor (Sigma, #SML0661) were resuspended in DMSO at 3 mM and 50 mM, respectively, and used at dilutions noted in the results section.

### 2.3. Antibodies

CD19 mouse anti-human antibody (clone 33-6-6, gift from Dr. Tom Tedder, Duke University, Durham, NC, USA) conjugated with either allophycocyanin or phycoerythrin (APC) was used as a surface B cell marker in flow cytometry. Mouse anti-human CD19 PE-Cyanine 7 antibody (eBioscience, San Diego, CA, USA, #25-0199-42) was used as an additional marker of the B cell surface. B cell surface markers were used at 1 μL per 10^6^ cells. PML antibody (Santa Cruz Biotechnology, Dallas, TX, USA, #sc-966) was used at 1:50 to detect PML expression by immunofluorescence. 53BP1 (Cell Signaling Technology, Danvers, MA, USA, #4937) and phosphorylated γH2AX (S139) (Cell Signaling Technology, #9817) were used as markers of DNA damage for immunofluorescence at 1:50. TelC-FITC (PNA Bio, Newbury Park, CA, USA #F1009) is a telomere PNA probe used as discussed below for immunofluorescence-FISH to detect the C-rich leading strand.

### 2.4. Infections and Cell Sorting

PBMCs were isolated from a buffy coat and stained with CellTrace Violet (Invitrogen, Carlsbad, CA, USA #C34557) using the manufacturer’s suggested protocol followed by infection with EBV at an MOI of 5 (such that all infected B cells are positive for EBNA-LP). On day 4 post-infection, PBMCs were stained with CFSE (Sigma, #21888) using the manufacturer’s suggested protocol. Proliferation was monitored in CD19+ B cells by the dilution of the CellTrace Violet and CFSE stains for up to eight days post-infection on a BD FACS Canto II and analyzed using FlowJo 10.0 software (TreeStar) (FlowJo, Ashland, OR, USA). On day 8 the arrested, CD19-positive cells were sorted based upon their diluted CellTrace Violet and high CFSE profile using either a Beckman Coulter Astrios or Beckman Coulter MoFlo XDP sorter (Beckman Coulter, Brea, CA, USA).

### 2.5. Immunofluorescence

EBV B95-8-infected B cells were pelleted, resuspended in 25 μL of PBS, spread on a microscope slide, and dried at 37 °C for 15 min. Cells were fixed in 4% paraformaldehyde for 15 min at 4 °C, washed in PBS, permeablized in PBS containing 0.2% Triton X-100 for 10 min, and then blocked in PBS with 0.2% Triton X-100 containing 5% normal goat serum for 1 h. Primary antibodies were incubated overnight at 4 °C followed by secondary antibody incubation with AlexaFluor 488 goat anti-rabbit IgG (Life Technologies, Carlsbad, CA, USA, #A11034) and AlexaFluor 568 goat anti-mouse IgG (Life Technologies, #A11004) for 2 h. Slides were mounted in Vectashield containing DAPI (Vector Laboratories, Burlingame, CA, USA, #H-1200). All immunofluorescence slides were visualized using a Zeiss 780 upright confocal microscope (Zeiss, Oberkochen, Germany) and images were analyzed using ImageJ version 2.0 (https://imagej.net/Welcome).

### 2.6. Immunofluorescence-Fluorescence In Situ Hybridization

EBV B95-8-infected B cells were pelleted, resuspended in 25 μL of PBS, spread on a microscope slide, and dried at 37 °C for 15 min. Cells were fixed in 4% paraformaldehyde for 15 min at 4 °C, washed in PBS and permeablized in PBS containing 0.2% Triton X-100 for 10 min. Slides were denatured at 77.8 °C for 5 min in a hybridization mix composed of 10 mM Tris-HCl (pH 7.5), 70% formamide, 0.5% blocking reagent (Roche, Basel, Switzerland, #11096176001), and 0.2–0.5 μg/mL PNA-TelC probe, in ddH20. After denaturation, slides were incubated overnight at 37 °C. Slides were washed first in wash buffer containing 70% formamide, 10 mM Tris pH7.5 and 0.1% BSA two times for 15 min, followed by a wash in 4X saline-sodium citrate (SSC) buffer containing 0.05% Tween-20 for 5 min and blocked for 15 min in 1X Roche blocking reagent diluted in 4X SSC. Primary antibodies were incubated overnight at 4 °C followed by secondary antibody incubation with AlexaFluor 488 goat anti-rabbit IgG (Life Technologies, #A11034) and AlexaFluor 568 goat anti-mouse IgG (Life Technologies, #A11004) for 2 h. Slides were mounted in Vectashield containing DAPI (Vector Laboratories, #H-1200). All slides were visualized using a Zeiss 780 upright confocal microscope and images were analyzed using ImageJ version 2.0. Co-localization of TelC with γH2AX was determined by calculating the Pearson’s coefficient using JACoP plugin for ImageJ. Based on average Pearson’s coefficient values for controls, including LCLs and bleomycin-treated LCLs, we defined TIF positive cells as cells with a Pearson’s coefficient above 0.35.

### 2.7. Cell Proliferation Assays

**Figure 1:**
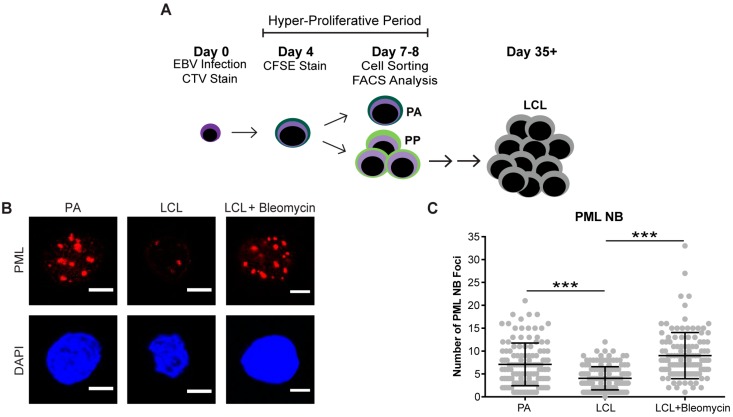

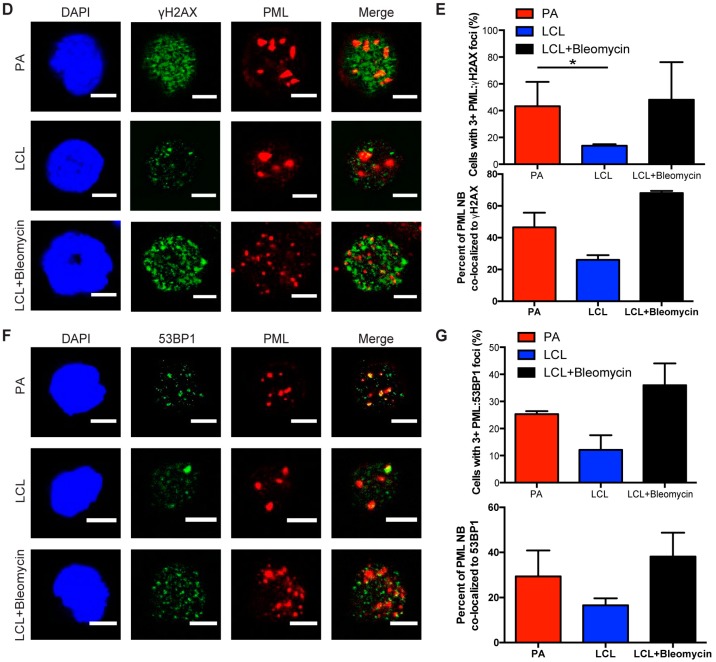
Persistent DNA damage foci increase in arrested EBV-infected B cells. (**A**) Schematic demonstrating infection, staining and sorting protocol to separate early, hyper-proliferating and then arrested (PA) cell populations from proliferating populations (PP), along with the generation of immortalized LCLs. (**B**) Immunofluorescence (IF) of PML NBs (red) and DAPI (blue) measured from sorted arrested B cells (PA), LCLs, and bleomycin-treated LCLs. (**C**) Quantification of PML NB foci per nucleus from (**A**). Error bars represent SD of three independent donors. *** *p* < 0.001 as determined by a Mann-Whitney test. (**D**) IF of γH2AX (green), PML NBs (red), and DAPI (blue) measured from sorted arrested B cells, LCLs, and bleomycin-treated LCLs. Co-localization of γH2AX-PML is shown in Merge. (**E**) Upper, quantification of cells with three or more PML NBs co-localized with γH2AX per nucleus from (**D**). Lower, quantification of percent γH2AX co-localization with PML per cell from (**D**). Error bars represent S.E.M of three independent donors for PA and LCL and two donors for LCL plus bleomycin. * *p* < 0.05, *** *p* < 0.001 as determined by a Student’s *t*-test. (**F**) IF of 53BP1 (green), PML NBs (red), and DAPI (blue) measured from sorted arrested B cells, LCLs, and bleomycin-treated LCLs. Co-localization of 53BP1-PML is shown in Merge. Note: the single, large 53BP1 focus co-localizing with PML is likely an Oct-1, PTF, transcription (OPT) domain [37]. (**G**) Upper, quantification of cells with three or more PML NBs co-localized with 53BP1 per nucleus from (**F**). Lower, quantification of percent 53BP1 co-localized with PML per cell from (**F**). Error bars represent S.E.M. of two independent donors. All scale bars indicate 5 μm.

PBMCs were infected on Day 0, stained with CellTrace Violet, and treated with either 3 μM of Danazol or 12 μM, 50 μM, or 100 μM of ML216 inhibitor. Cells were put back into culture and proliferation was monitored for CD19+ B cells by the dilution of the CellTrace Violet stain at day 8 post-infection using a BD FACS Canto II. Early proliferating B cells, which include both the hyper-proliferating and then arrested (PA) and proliferating populations (PP) populations in bulk, are comprised of population doublings (PD) 1–4 (Figure 1A).

### 2.8. Transformation Assay

PBMC infection was conducted in the presence of 0.1% DMSO and 3 μM danazol added at day 0 post-infection. Supernatant from B95-8 Z-HT cells was titrated from 300 μL/10^7^ PBMCs to 0.03 μL/10^7^ PBMCs. Each infection point consisted of 7 × 10^6^ infected PBMCs seeded in 20 wells of a 96-well plate. The percentage of wells positive for B cell outgrowth into LCLs at five weeks post infection was plotted relative to the multiplicity of infection (MOI) per well. The efficiency of transformation was determined as published where 1 transforming unit per well was considered the amount of B95-8 virus necessary to yield 62.5% of positive wells [3].

### 2.9. Real-Time qPCR

Total RNA was isolated by using RNeasy (Qiagen, Hilden, Germany, #74106) and reverse-transcribed by using a High Capacity cDNA Reverse Transcription kit (Life Technologies, #4368814) according to the manufacturer’s instructions. Relative mRNA abundance was measured by using SYBR green-based RT-qPCR assay with 5 ng of cDNA per reaction. All primers (IDT, Coralville, IA, USA) were used at 1 μM per reaction. qRT-PCR was performed using the StepOnePlus Real-Time PCR light-cycler (Applied Biosystems, Foster City, CA, USA) and analyzed by using the StepOne software (version 2.3, Applied Biosystems). All samples were analyzed in triplicate and expression levels were normalized, first, to SET Domain Bifurcated 1 (SETDB1), and then to DMSO treated controls. Primer sequences for RT-qPCR were as follows: hTERT: forward primer, 5′-CCGATTGTGAACATGGACTACG-3′, and reverse primer, 5′-CACGCTGAACAGTGCCTTC-3′ and SETDB1: forward primer, 5′-TCCATGGCATGCTGGAGCGG-3′, and reverse primer, 5′-GAGAGGGTTCTTGCCCCGG-3′.

## 3. Results

### 3.1. EBV Infection of Primary Human B Cells Induces an Increase in PML NBs and Association with DDR Foci

Cellular senescence often occurs when a cell undergoes DNA damage that cannot be repaired. In the context of irreparable DNA damage, the DDR signaling pathway is persistently activated, which leads to the formation of persistent DNA damage foci and promotes establishment of senescence. To determine whether hallmarks of a persistent DDR are present in EBV-infected B cells that undergo senescence we sought to specifically examine the early subpopulation of B cells that arrest upon hyper-proliferation. We used a double-staining technique previously developed by our lab to sort infected B cells that proliferated and then arrested (PA) from cells that become indefinitely-proliferating LCLs [24]. Initially, we stained bulk PBMCs with CellTrace Violet (CTV) and infected the cells with EBV at a multiplicity of infection (MOI) such that every CD19+ B cell is latently infected [5]. On day 4 post-infection, we applied a second proliferation tracking dye, carboxyfluorescein succinimidyl ester (CFSE), to the cells and returned them back to culture. At eight days post-infection, we sorted CD19-positive EBV-infected B cells that arrested based on diluted CTV and high CFSE fluorescence profiles (Figure 1A). Our goal in these studies is to characterize the nature of the DDR, which is enriched in the PA population. Therefore, we focused on PA and not PP cells, and we also examined immortalized LCLs as a negative control and LCLs treated with high dose bleomycin sulfate as a positive control for persistent DDR [35]. We first assayed these cell populations for the major focal component of persistent DDR foci, PML NBs, and found that arrested cells exhibited a significantly higher number of PML NB foci per nucleus as compared to LCLs. This similar increase in PML NBs was also observed in LCLs treated with bleomycin (Figure 1B,C). Furthermore, we investigated whether PML NBs co-localized to markers of DNA damage, a characteristic of persistent DDR foci, and found that arrested cells and LCLs treated with bleomycin exhibited an increase in PML NBs co-localized to DDR markers, including γH2AX and 53BP1 (Figure 1D–G). These data suggest that arrested EBV-infected B cells induce an increase in PML NBs that associate with DNA damage to form a key feature of persistent DDR foci.

### 3.2. Persistent DNA Damage Response Is Localized to Telomeric DNA in Early Arrested EBV-Infected B Cells

**Figure 2:**
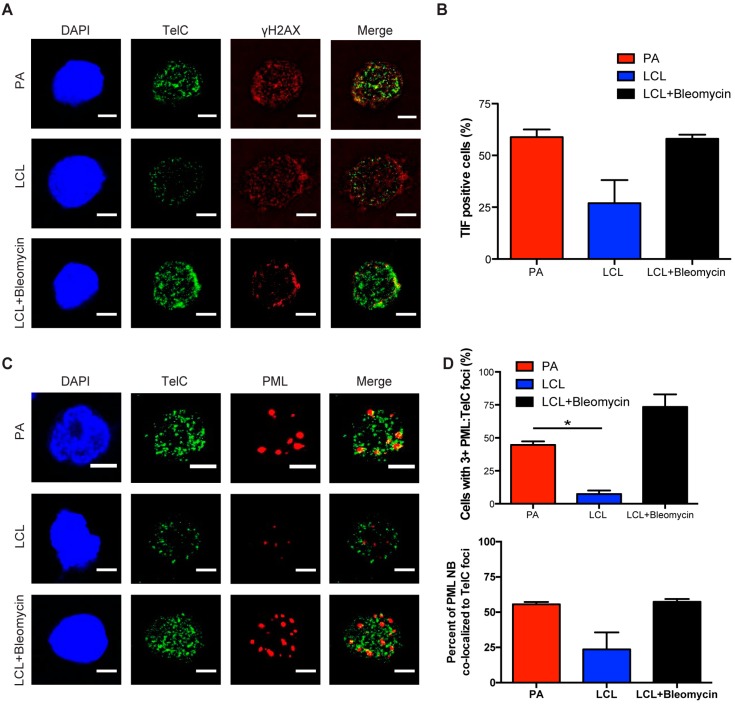
Persistent DNA damage localizes to telomeres in arrested EBV-infected B cells. (**A**) IF-telomere FISH of γH2AX (red), TelC (green), and DAPI (blue) measured from sorted arrested B cells, LCLs, and bleomycin-treated LCLs. Co-localization of γH2AX-TelC is shown in Merge. (**B**) Quantification of TIF positive cells as defined by the co-localization of TelC with γH2AX from (**A**). (**C**) IF-telomere FISH of PML NBs (red), TelC (green), and DAPI (blue) measured from sorted arrested B cells, LCLs, and bleomycin-treated LCLs. Co-localization of PML-TelC is shown in Merge. (**D**) Upper, quantification of cells with greater than three PML NBs co-localized with TelC per nucleus from (**C**). Lower, quantification of the percentage of PML NBs co-localized to TelC foci per cell. Error bars represent S.E.M of three independent donors. * *p* < 0.05 as determined by a Student’s *t*-test. For lower graph, PA vs. LCL *p* = 0.0576. All scale bars indicate 5 μm.

Persistent DDR foci have been shown by a number of groups to preferentially target sites of irreparable DNA damage including telomeres [32,33]. We have observed characteristics of persistent DNA damage foci in the arrested population of EBV-infected B cells and sought to further investigate whether these foci specifically form at sites of telomeric DNA damage upon infection. We conducted immunofluorescence-telomere fluorescence in situ hybridization (IF-Telomere FISH) to examine the localization of DDR markers to telomeric DNA, also known as telomere dysfunction-induced foci (TIF). To label telomeres we used a TelC PNA FISH probe that is a C-rich probe, which recognizes the leading strand TAACCC repeats. We found that arrested infected B cells exhibited an increase in TIF positive cells as measured by the co-localization of TelC to γH2AX, while LCLs exhibited reduced levels of TIFs (Figure 2A,B).

Next we wanted to determine whether telomeres also localized to PML NBs, known as ALT-associated PML NBs (APBs), a primary characteristic of persistent DDR foci. Recently, the Masucci group showed that bulk early-infected B cells activated the non-canonical telomere maintenance pathway, alternative lengthening of telomeres (ALT). In doing so, they examined the state of telomere dysfunction in infected B cells and reported an increase in the presence of APBs [34]. Here we specifically study the arrested subpopulation of early EBV-infected B cells and consistent with their findings, we observed a significant increase in the presence of PML NBs co-localized to telomeric DNA as compared to LCLs (Figure 2C,D). Together, these findings suggest that arrested EBV-infected B cells exhibit characteristic markers of persistent DDR foci that accumulate at telomeric DNA suggesting that telomere dysfunction contributes to the establishment of OIS mediated by EBV infection.

### 3.3. Increased hTERT Expression Enhances EBV-Mediated Transformation of Early-Infected B Cells

**Figure 3:**
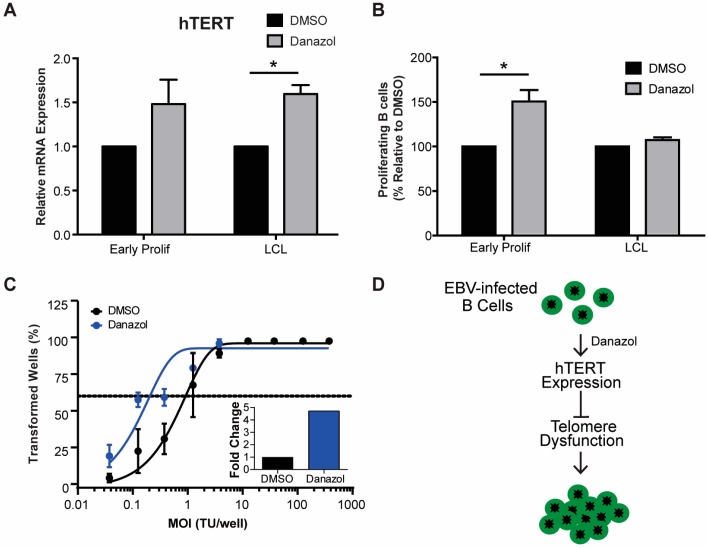
Danazol treatment enhances EBV-mediated B-cell transformation. (**A**) The expression level of hTERT mRNA was measured from sorted early proliferating CD19+ infected B cells on day 7 and LCLs. Relative mRNA abundance was normalized to SETDB1. Data are represented as the fold change relative to DMSO treatment. Error bars represent S.E.M. of three independent donors. * *p* < 0.05 as determined by a Student’s *t*-test. (**B**) Percentage of proliferating CD19+ B cells was determined for early proliferating infected B cells and LCLs that were treated with DMSO (black) or with 3 μM danazol (grey) at the time of infection. The data were analyzed by FACS at day 7 post-infection. Error bars represent S.E.M. of three independent donors. * *p* < 0.05 as determined by a Student’s *t*-test. (**C**) Quantification of EBV-infected B cell outgrowth following PBMC infection in the presence of DMSO (black) or 3 μM danazol (blue) at the time of infection. The percentages of wells positive for LCLs at five weeks post-infection are plotted relative to the transforming units (TU) of B95-8 virus per well. Error bars represent S.E.M of three independent donors. Dotted line represents 62.5% positive wells, which indicates outgrowth from the virus amount in the *x*-axis of a single LCL per well based on a Poisson’s distribution. (**C**, **inset**) Fold change of the transformation efficiency. (**D**) Schematic of danazol’s mechanism of action during EBV infection.

Oncogenic signaling has been shown to play a major role in senescence by inducing telomeric replication stress and telomere dysfunction in cells that lack sufficient hTERT activity [38]. Importantly, while telomeric repeats are hypersensitive to DNA replication stress it has been reported that hTERT expression can mitigate telomere dysfunction [38]. Since primary human B cells are intractable for heterologous over-expression studies, we sought to use a pharmacological approach to determine if increased hTERT expression can allow early-infected B cells to overcome TIF-associated growth arrest. Recent evidence suggests that androgen hormones can promote hTERT expression and, in fact, danazol has recently been described as a new therapy for patients with telomere diseases [39,40]. Addition of danazol to bulk EBV-infected early, proliferating B cells (population doubling 1–4) and LCLs increased the mRNA level of hTERT (Figure 3A). We, therefore, assessed whether hTERT upregulation would impact transformation as early-infected cells displayed increased TIFs. Treatment of PBMCs with 3 μM danazol concurrent with EBV infection led to an increase in the number of CD19+ proliferating B cells at day 7 post-infection relative to untreated cells (Figure 3B). However, treatment of LCLs with danazol had no effect on cell proliferation, thus suggesting that danazol acts on a process only relevant early after infection (Figure 3B). Furthermore, we observed an increase in EBV-mediated transformation efficiency with danazol treatment relative to DMSO-treated infected PBMCs (Figure 3C). Collectively, these findings support a model whereby defective telomere maintenance contributes to the arrest of early proliferating B cells and ultimately suppresses EBV-mediated transformation (Figure 3D).

### 3.4. Early EBV-Infected B Cells Are Sensitive to Inhibition of BLM Helicase

**Figure 4:**
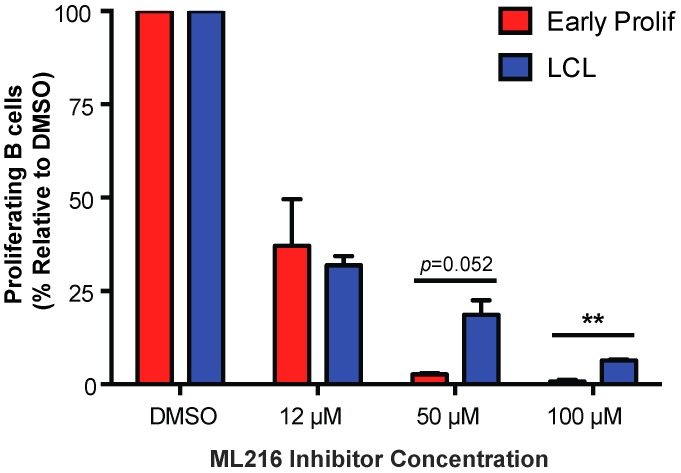
Early proliferating EBV-infected B cells are more sensitive to BLM helicase inhibition than LCLs. Percentage of proliferating CD19+ B cells was determined for early proliferating EBV-infected B cells (red) and LCLs (blue) that were treated with DMSO or with 12 μM, 50 μM, or 100 μM ML216 BLM helicase inhibitor at the time of infection. The data were analyzed by FACS at day 7 post-infection. Error bars represent S.E.M. of three independent donors. ** *p* < 0.01 as determined by a Student’s *t*-test.

To further investigate the role of telomere maintenance in establishment of a persistent DDR we examined the importance of Bloom syndrome (BLM) helicase in early infected B cells. BLM helicase is a member of the RecQ helicase family, which is involved in homologous recombination and is capable of unwinding G-quadruplex DNA structures formed by telomeric DNA [41,42,43]. BLM helicase is specifically important for cells that undergo alternative lengthening of telomeres (ALT) rather than the telomerase-dependent cannonical method of telomere maintenance [44]. In a recent report, it has been shown that EBV-infected B cells maintain telomeres via the ALT pathway [34]. To study the importance of BLM helicase in regulating telomere maintenance during early EBV infection we, again, turned to a pharmacological approach using ML216, a small molecule inhibitor of BLM [43]. We found that early proliferating EBV-infected B cells are more sensitive to BLM helicase inhibition by ML216 than LCLs (Figure 4). These data suggest that inhibiting telomere maintenance factors in cells that are already undergoing replicative stress may exacerbate the telomere dysfunction causing cells to become more sensitive to persistent DDR.

## 4. Discussion

The DDR signaling pathway is known to be an important innate tumor suppressor pathway involved in repairing damaged DNA, inducing apoptosis or arresting the cell cycle. DNA damage at irreparable sites can facilitate entry into an irreversible growth arrest. Importantly, persistent activation of the DDR has been causally linked to the establishment of senescence [31,32,33,35]. We have previously found that EBV infection in vitro induces a transient period of hyper-proliferation early after infection leading to initiation of DDR-mediated senescence [5]. Furthermore, we observed metabolic stress and presence of limited nucleotide pools that were insufficient to overcome rapid proliferation contributing to maintenance of OIS in EBV-infected B cells [9,24]. We propose that DNA damage and replicative stress sustained early during hyper-proliferation mediates the formation of persistent DNA damage foci in early EBV-infected B cells to maintain senescence. In this study, we sought to identify the presence of persistent DDR foci and characterize these foci specifically in the arrested subpopulation of EBV-infected B cells. We found that, upon EBV infection, the arrested B cells exhibited an increase in PML NBs as well as PML NB-associated DNA damage foci. We went on to explore the state of telomeres in the arrested infected B cells and observed that persistent DNA damage localized to telomeric DNA forming TIFs. Intriguingly, a drug used to treat patients with telomere maintenance disorders, danazol, was found to increase the proliferation of B cells specifically early after infection and enhance transformation of B cells into LCLs. Lastly, we found that early proliferating B cells are more sensitive than LCLs to inhibition of a key telomere replication protein, BLM helicase.

Persistent DDR signaling has been previously shown to form unique persistent DNA damage foci in cells undergoing OIS. The Campisi laboratory has extensively investigated the spatiotemporal dynamics of persistent DNA damage foci and established a role for chronic DDR signaling in maintenance of senescence [35]. They report that PML NBs are an important staple to the formation of persistent DNA damage foci, which associate with both γH2AX and 53BP1 DDR markers, as well as telomeres. Additionally, Bazett-Jones and colleagues have previously shown that PML NBs act as DNA damage sensors and increase in number with the activation of the DDR [45]. As DNA damage accumulates it alters the chromatin state and causes PML NBs to form microbodies [46]. The characteristics we describe here of PML-associated DNA damage foci are consistent with these findings, and further support a role for PML NBs serving as a potential scaffold for persistent DNA damage foci responsible for facilitating senescence.

Reparable DNA damage leads to the formation of transient DNA damage foci that are typically resolved in 24 h. However, in the case of irreparable DNA lesions, persistent DNA damage foci are formed allowing for the maintenance of irreversible senescence. Telomeric DNA has been established by many groups to be a primary site of irreparable damage and, therefore, is a favored target of a persistent DNA damage response [32,33]. Here, we have shown that telomeric DNA is targeted by markers of DNA damage and that persistent DNA damage foci are localized to telomeres in arrested EBV-infected B cells. These findings are consistent with an array of evidence in the senescence field. d’Adda di Fagagna and colleagues have laid much of the foundation for these findings and have specifically shown that persistent DDR is a mechanism for mediating senescence and that telomeric DNA damage is irreparable and associated with persistent DDR signaling [31,32,33,35]. Utz Herbig’s group has recently demonstrated that replicative stress and irreparable telomeric DNA damage mediate OIS [38]. Importantly, Masucci’s laboratory has recently shown that early proliferating EBV-infected B cells exhibit a range of telomere dysfunction, including the localization of PML NBs to telomeric DNA. Consistent with these findings, our report goes on to show that the arrested subpopulation of early EBV-infected B cells exhibit telomere dysfunction and telomeric-associated persistent DNA damage foci.

Telomere maintenance is important for proper protection and replication of telomeric DNA. Genetic defects in telomere maintenance and repair have been shown to result in telomere diseases, including bone marrow failure, liver cirrhosis, and increased risk of cancer. Specifically, deficient hTERT activity is known to induce telomere dysfunction and persistent DDR localized to telomeres [38]. Telomere dysfunction occurs early, within days, in EBV-infected B cells, therefore, it is unlikely that telomere abnormalities exist due to telomere shortening, but rather improper telomere maintenance, leading to telomere deprotection. Danazol, an androgen hormone recently tested in clinical trials for the treatment of telomere diseases has been shown to elongate telomeres in patients with telomere diseases and to have a greater impact on patients with *TERT* mutations [40]. The mechanism behind how danazol elongates telomeres is not well understood; however, evidence has been shown to suggest that androgen therapies have a direct effect on telomerase activity by upregulating hTERT expression [39]. In line with this mechanism we have shown here that danazol increases hTERT mRNA expression in EBV-infected B cells. As danazol treatment also promotes infected B cells to overcome cellular arrest and enhances transformation efficiency it is possible that hTERT expression is deficient in early infected cells. Furthermore, we found that early EBV-infected B cells displayed elevated sensitivity to ML216, an inhibitor of BLM helicase, which is involved in telomere replication [41,42,43,44]. This further implicates telomere maintenance in the establishment of persistent DDR foci and senescence in early EBV-infected B cells.

In summary, EBV infection induces a period of rapid cell proliferation (~8–12-h per cell cycle) that presents a challenge for proper DNA replication. In the context of metabolic stress and insufficient nucleotide pools, most infected B cells fail to be immortalized by EBV, but rather undergo OIS due to a persistent DNA damage response. Our group, corroborating initial findings by Masucci, provide data supporting a model whereby irreparable DNA damage at telomeres is the molecular source for the OIS-mediated DDR. Future studies will be aimed at defining the role of telomere dysfunction and the early period of hyper-proliferation in the restriction and development of EBV-associated lymphomas in vivo.
